# Supplementary material for: CCN1 is an opsonin for bacterial clearance and a direct activator of Toll-like receptor signaling
Source: Nat Commun. 2020 Mar 6;11:1242. doi: 10.1038/s41467-020-15075-5 (PMC7060279; doi:10.1038/s41467-020-15075-5)
Supplement: Supplementary file 1 — Supplementary Information [file 41467_2020_15075_MOESM1_ESM.pdf]

Supplementary Information

CCN1 is an opsonin for bacterial clearance and a direct activator of Toll-like receptor signaling

Jun and Lau

.

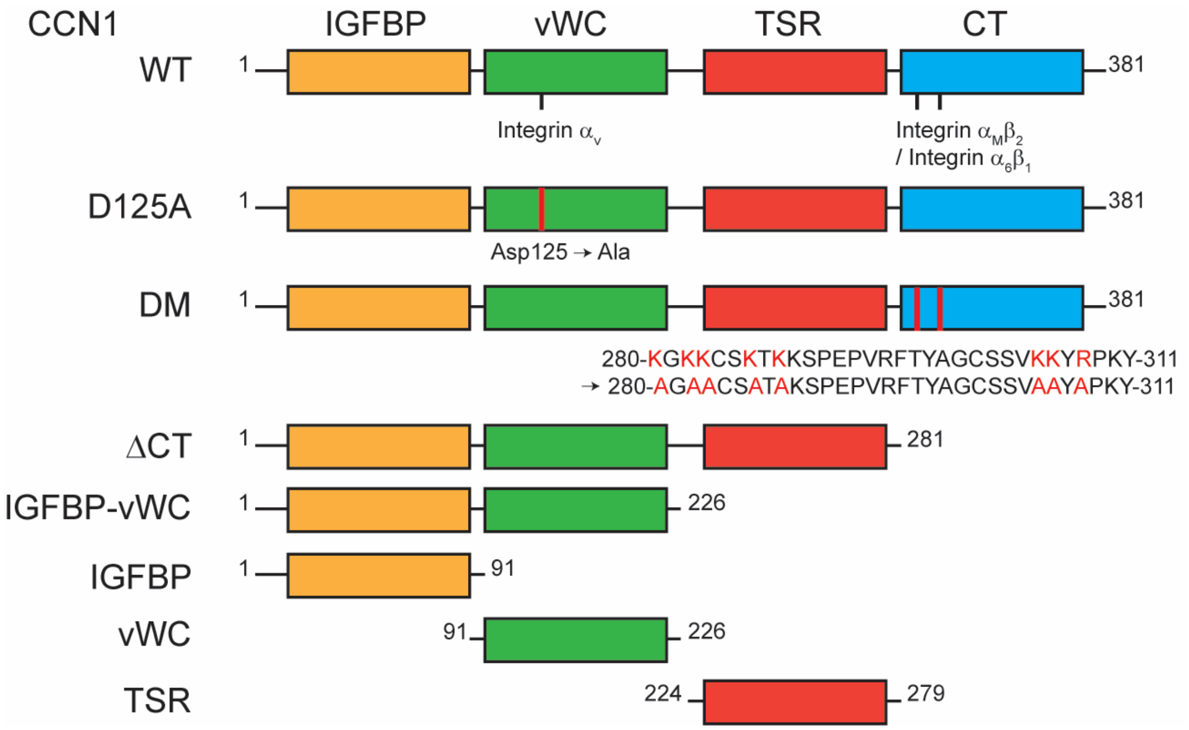

Supplementary Figure 1. Schematic diagram of CCN1 and mutant proteins. CCN1 contains 4 domains with homologies to insulin-like growth factor binding protein (IGFBP; yellow), von Willebrand factor type-C repeat (vWC; green), thrombospondin type-1 repeat (TSR; red), and the cysteine-knot motif (CT; blue). Integrin binding sites are marked. D125A is an integrin $\alpha_v\beta_3$ binding defective mutant with Asp125 to Ala substitution in the vWC domain (red line). DM is an integrins $\alpha_M\beta_2/\alpha_6\beta_1$-binding defective mutant with two clusters of mutation sites (red lines) in the CT domain. Specific amino acid changes are shown. ΔCT, deletion of the CT domain; IGFBP-vWC, deletion mutant containing only the first two domains. IGFBP, IGFBP domain alone; TSR, TSR domain alone; vWC, vWC domain alone.

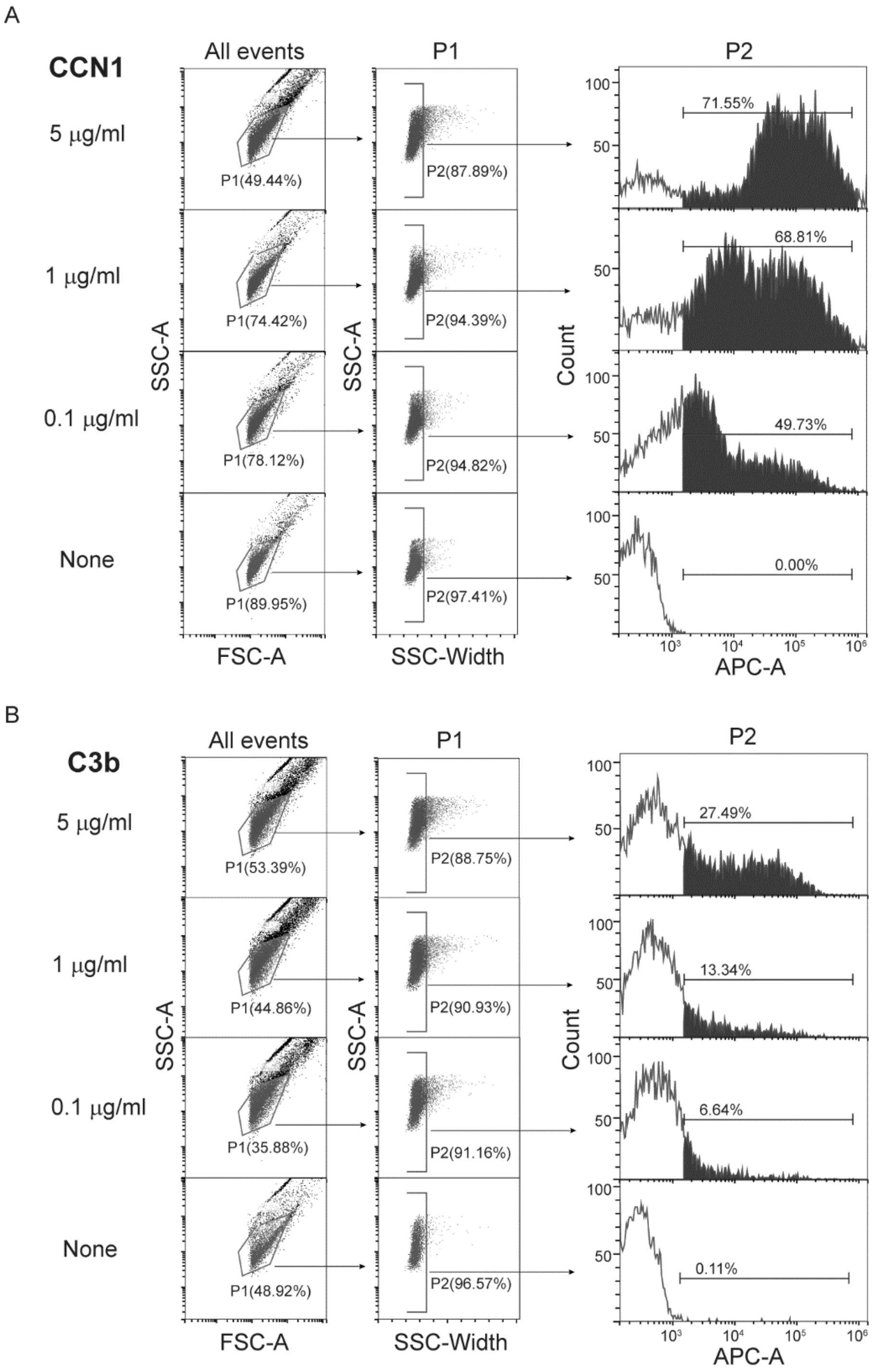

Supplementary Figure 2. CCN1 binding to *S. aureus* in flow cytometry. Heat-killed *S. aureus* (10^8 CFU) were incubated with increasing concentrations of CCN1 (**A**) or human C3b complement protein (**B**). *S. aureus* binding to CCN1 or C3b was measured by FACS analysis using polyclonal anti-CCN1 antibody with APC-conjugated goat anti-rabbit IgG and monoclonal anti-iC3b antibody with APC-conjugated rat anti-mouse IgG. Gating strategies are shown in FSC vs SSC (*left*; All events) and SSC vs SSC-Width dot plot (*middle*; P1) to sort the target population (P2). To determine the percentage of APC-positive population (*right*; shaded), the number of events (y-axis) were recorded at specific fluorescence intensity (x-axis). No protein treatment (None) was used as a negative control to define APC-positive population.

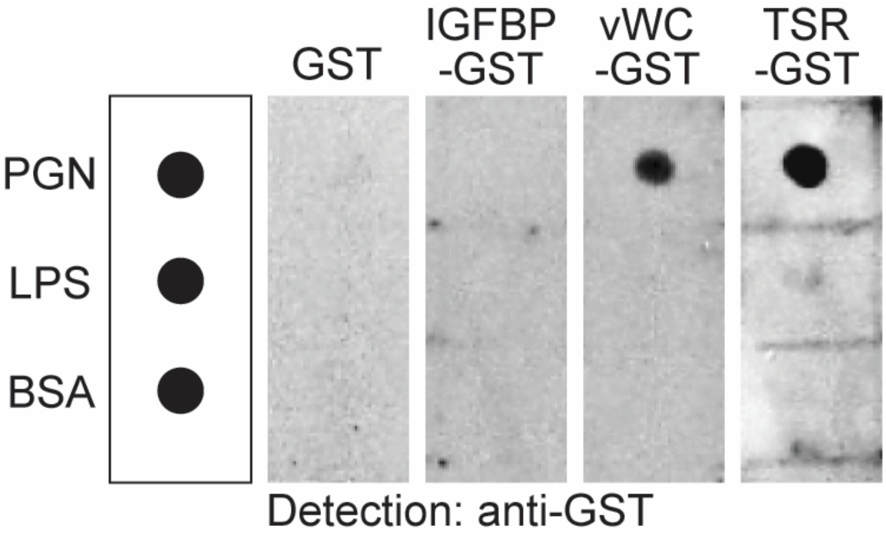

Supplementary Figure 3. PGN binding domains of CCN1. PGN from *S. aureus* and LPS from *P. aeruginosa* (1 µg each) were spotted onto nitrocellulose membranes and blocked with 2% PVA (vol per weight). BSA was used as control. GST protein and GST-CCN1 domain fusion constructs (IGFBP-GST, vWC-GST, TSR-GST; 2 µg each in PBS) were added and incubated for 4 h, and specific binding was visualized using anti-GST antibodies. The dot blot shown is a representative image.

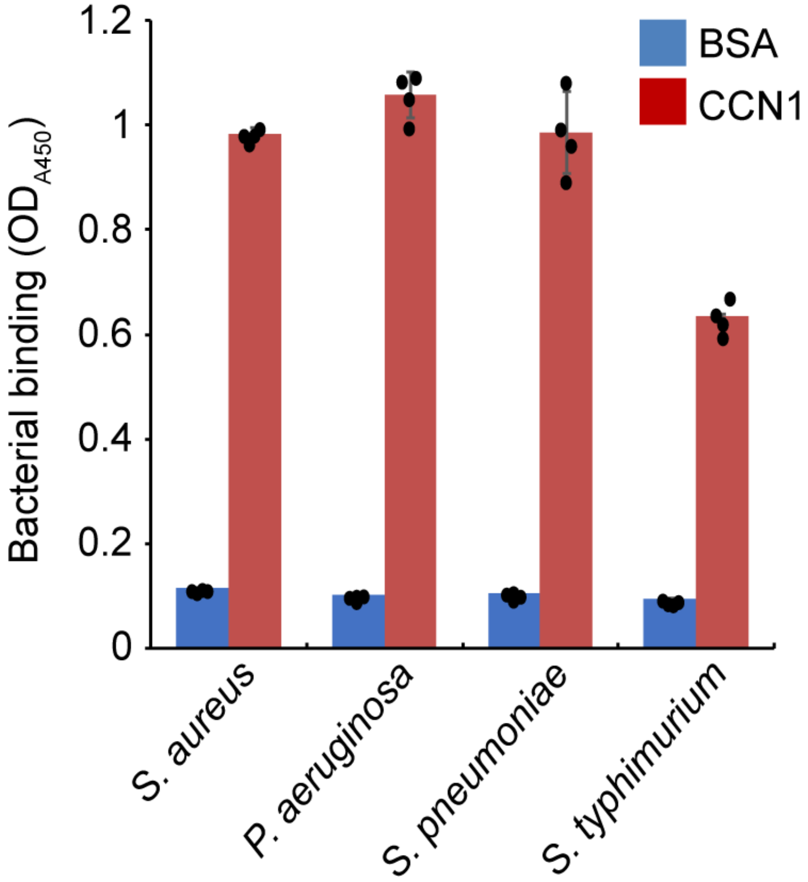

Supplementary Figure 4. CCN1 binds to various Gram-positive and Gram-negative bacteria. Solid-phase binding assays were performed with plates pre-coated with CCN1 (50 pmol per well) and incubated with distinct bacterial species, including the Gram-positive *S. aureus* and *S. pneumoniae,* and the Gram-negative *P. aeruginosa* and *S. typhimurium*. Data are expressed as mean ± s.d. in triplicate experiments. Source data are provided as a Source Data file.

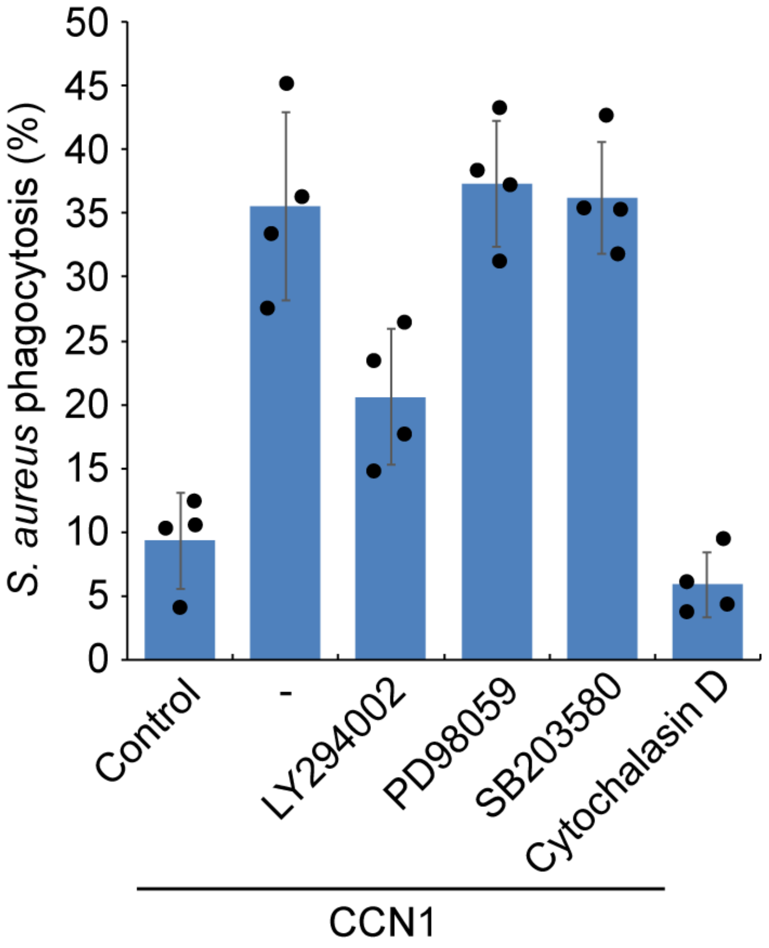

Supplementary Figure 5. Effects of chemical inhibitors on CCN1-induced phagocytosis. BMDMs were pretreated with CCN1 (2 μg per ml) with or without chemical inhibitors for 30 min, followed by incubation with *S. aureus* bioparticle (pHrodo® Red; 5 μg per ml) for an additional 40 min and measurement of phagocytosis. Chemical inhibitors used are cytochalasin D (actin polymerization inhibitor, 15 μM), LY294002 (PI3K inhibitor, 10 μM), PD98059 (ERK inhibitor, 10 μM), and SB203580 (p38 MAPK inhibitor, 10 μM). In control, BSA was added in lieu of protein or inhibitor. PI3K inhibitor partially blocked CCN1-induced phagocytosis, whereas cytochalasin D completely blocked it. Data are expressed as mean ± s.d. in triplicate experiments. Source data are provided as a Source Data file.

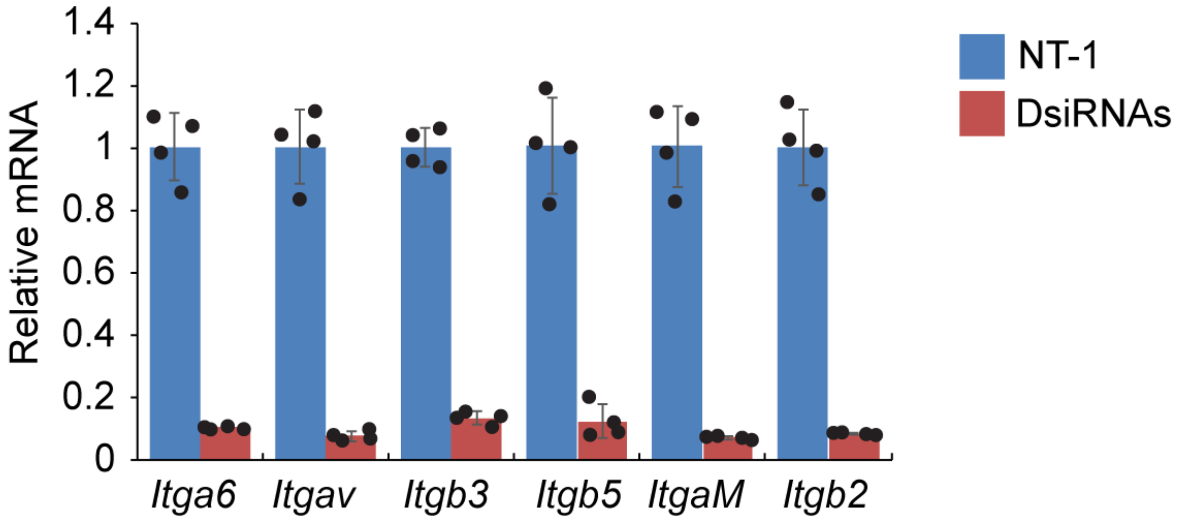

Supplementary Figure 6. DsiRNA-mediated knockdown of integrin subunits in BMDMs. BMDMs were transfected with predesigned DsiRNAs against individual integrin subunits and the knockdown efficiency was confirmed using qPCR analysis. All data are expressed as mean ± s.d. from triplicate determinations. Source data are provided as a Source Data file.

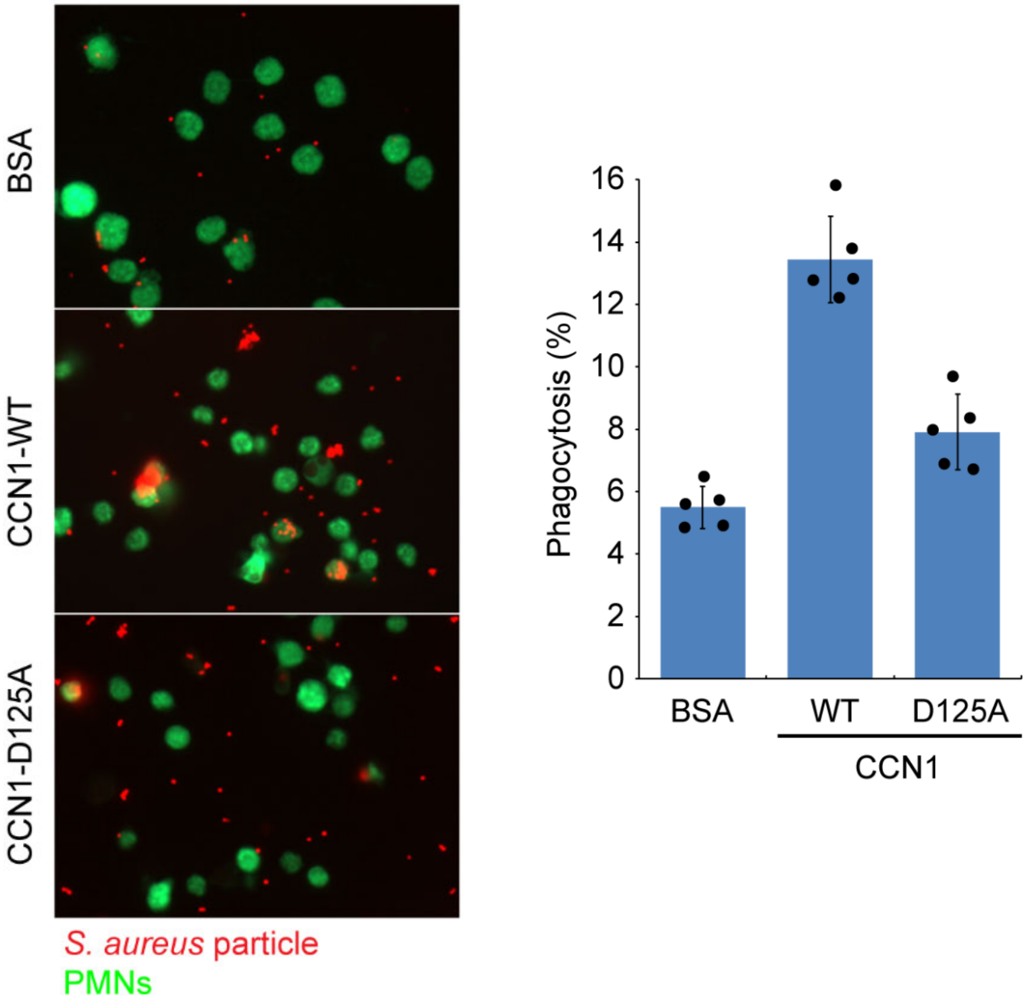

Supplementary Figure 7. CCN1-induced phagocytosis of *S. aureus* by PMNs. CCN1-induced phagocytosis of *S. aureus* was measured in PMNs. Cells were labeled with CellTracker-Green and then pretreated with either BSA, CCN1-WT, or CCN1-D125A mutant protein (2 µg per ml each) for 30 min, followed by incubation with *S. aureus* bioparticles (pHrodo® Red; 5 µg per ml) for 40 min. Phagocytosis was quantified under the fluorescence microscope by scoring ~300 cells in 10 independent images. Representative images are shown on left and quantification on right. Data are expressed as mean ± s.d. Source data are provided as a Source Data file.

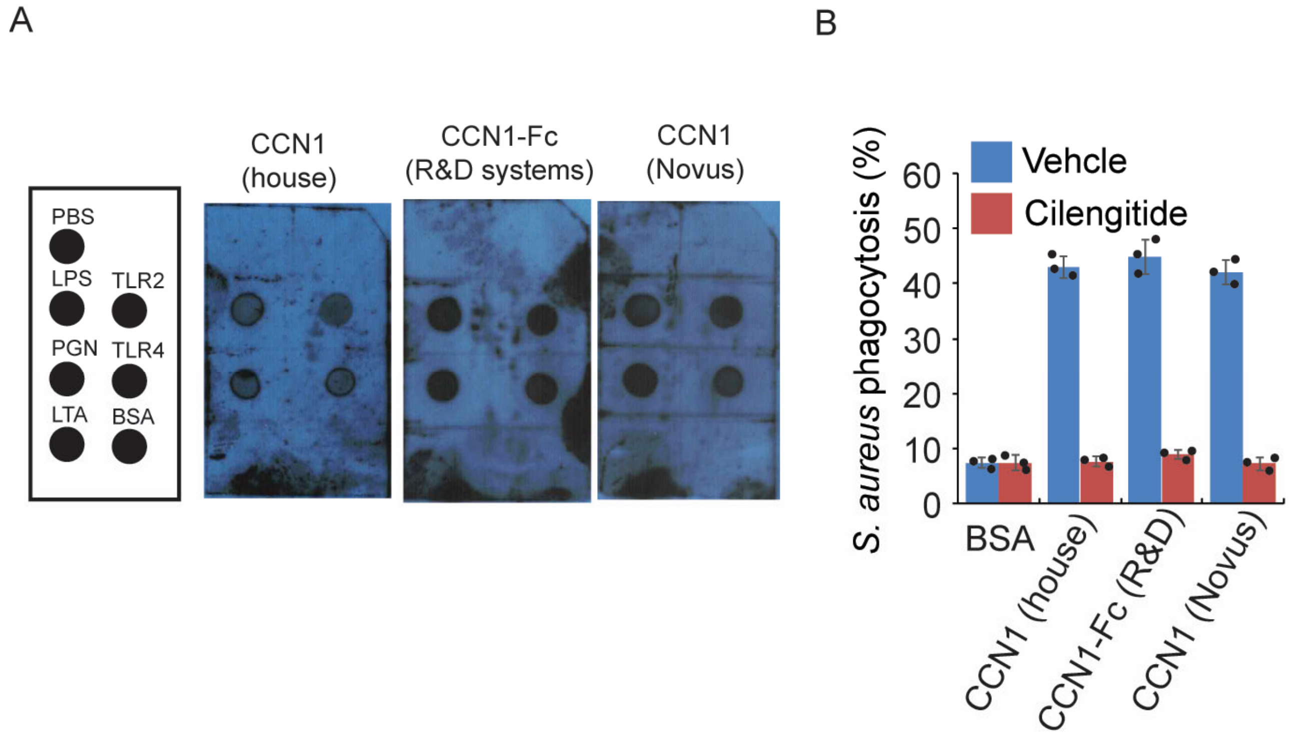

Supplementary Figure 8. Comparison of CCN1 protein from commercial sources.
Our house made CCN1 was compared to recombinant CCN1 protein from commercial sources. CCN1-Fc is a chimeric protein expressed in CHO cells (R&D biosystems, 4055-CR-050); CCN1 from Novus is expressed in *E. coli*. (Novus, NBP2-34944). (**A**) Dot blot assay. LPS, PGN, LTA, TLR2, TLR4, and BSA (1 µg each) were spotted on nitrocellulose membrane, incubated with CCN1 protein from various sources (2 µg each in PBS) and detected with anti-CCN1 antibodies. Along with CCN1 protein from house, CCN1-Fc and CCN1 from Novus also showed binding to bacterial pattern LPS and PGN as well as TLR2 and 4. Image is representative of 3 independent experiments. (**B**) House CCN1, CCN1-Fc and CCN1 from Novus (2 µg per ml each) all increased phagocytosis of *S. aureus* particles in BMDMs, an activity obliterated by cilengitide pretreatment (1µM). Data are expressed as mean ± s.d. from triplicate determinations. Source data are provided as a Source Data file.

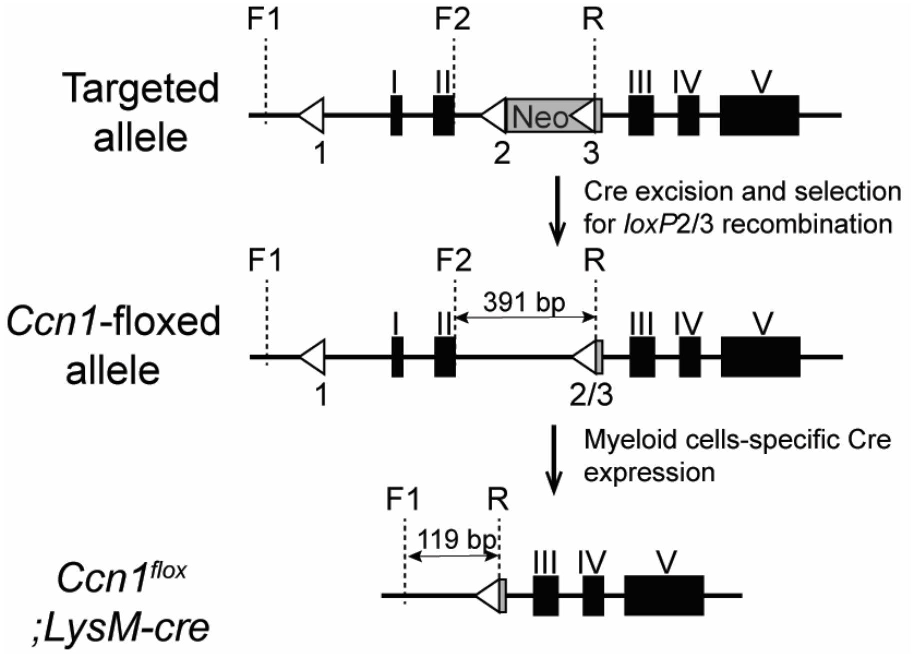

Supplementary Figure 9. Schematic diagrams showing the targeted *Ccn1* genomic locus with the *Ccn1*^{flox} allele and the neomycin resistance cassette (Neo). Left-pointing open triangles represent *loxP* sites. The neo marker was deleted by recombination between *loxP* sites 2 and 3, resulting in the *Ccn1*^{flox} allele. Recombination between *loxP* sites 1 and 2/3 leads to deletion of the first two exons of *Ccn1*, leading to a null allele.

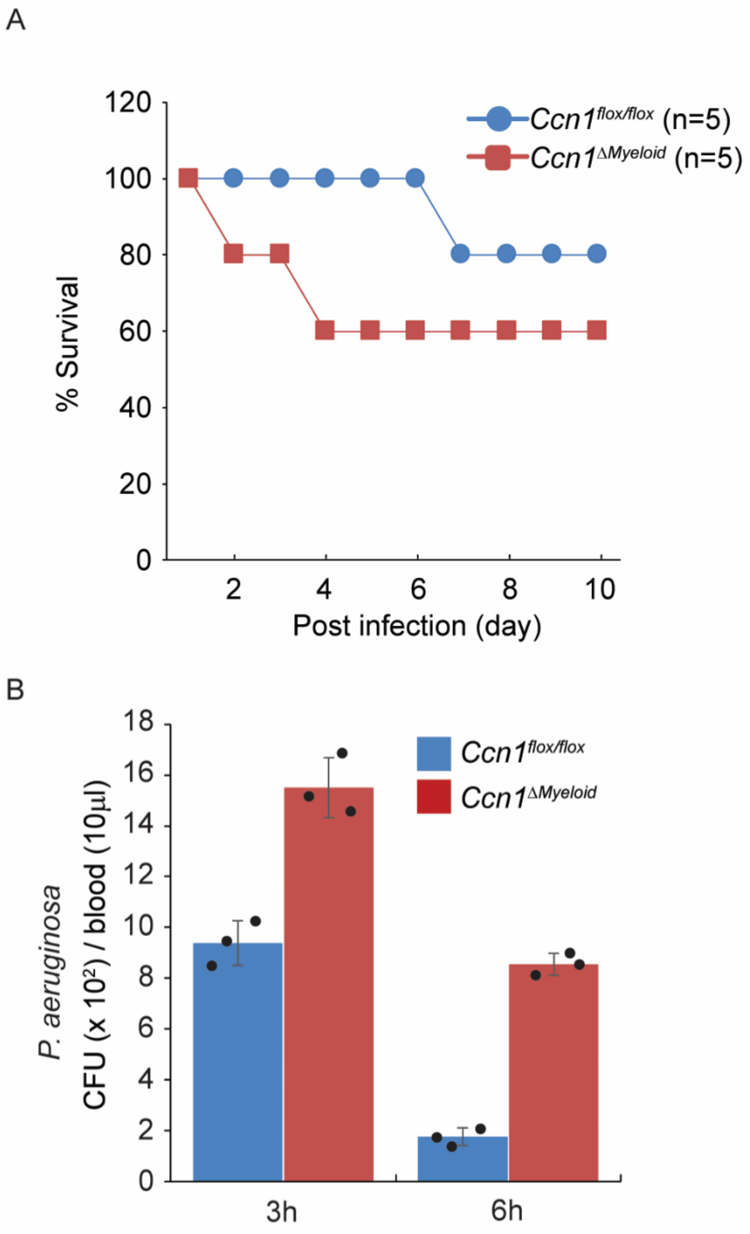

Supplementary Figure 10. ***P. aeruginosa*** **infection in mice.** (**A**) *P. aeruginosa* (2×10^8 CFU in 100 µl) were intravenously (*i.v.*) injected into the retro-orbital sinus of *Ccn1*$^{flox/flox}$ or *Ccn1*$^{\Delta Myeloid}$ mice. The survival was monitored over a 10-day period post-infection. (**B**) Acute peritonitis was induced by intraperitoneal (*i.p.*) injection of *P. aeruginosa* (5×10^7 CFU in 300 µl). Mice (n=3 each) were sacrificed at 3h and 6h. Blood (10 µl) drawn from mice were plated on tryptic soy agar plates with 5% sheep blood and viable bacteria were enumerated. Source data are provided as a Source Data file.

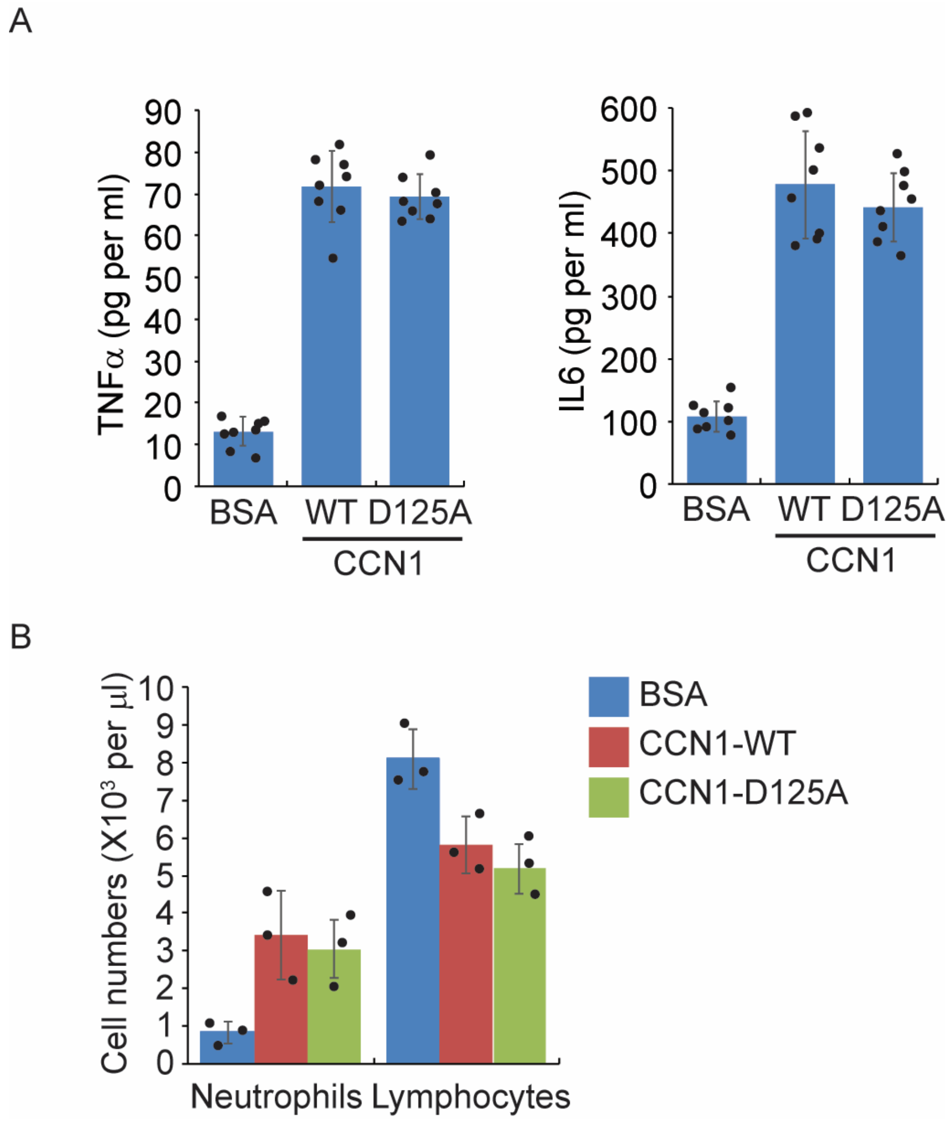

Supplementary Figure 11. CCN1 activates inflammatory responses in the absence of infection. C56BL/6J mice were *i.p.* injected with either BSA, CCN1-WT, or CCN1-D125A proteins (5 µg each, n=4 per group). (**A**) Peritoneal exudates were analyzed for TNFα at 30 min post-injection and IL6 at 2 h by ELISA. (**B**) CBC analysis was performed on blood from mice treated as above. Neutrophils and lymphocytes contents are shown. All data are expressed as mean ± s.d. from triplicate determinations. Source data are provided as a Source Data file.

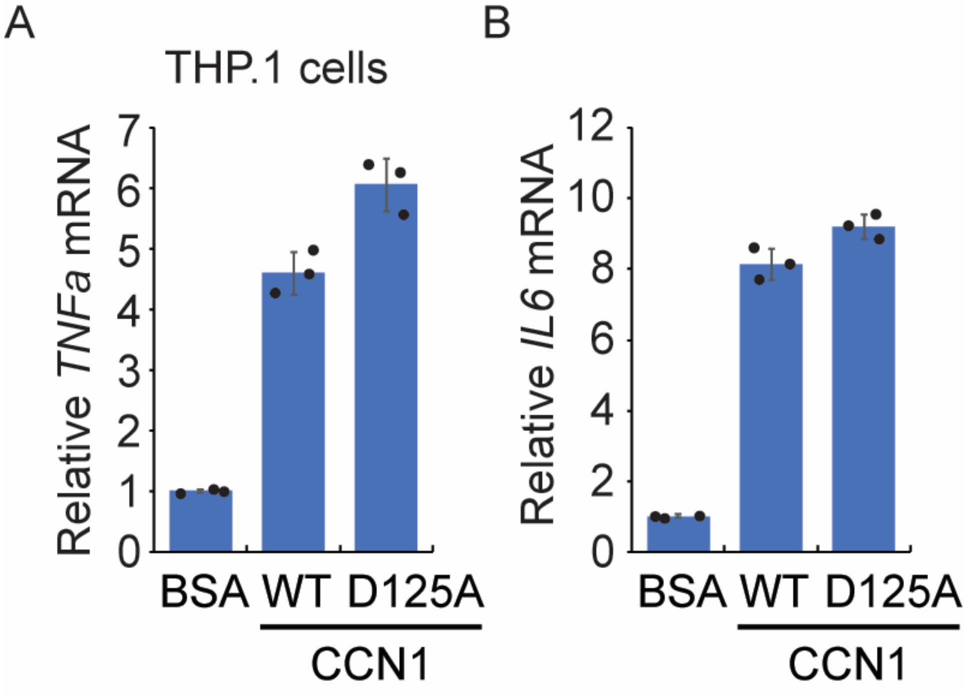

Supplementary Figure 12. CCN1 induces inflammatory response in human macrophages. Differentiated THP.1 human macrophage cell line was treated with either CCN1-WT or CCN1-D125A mutant proteins (2 µg per ml each) for 6 h. qPCR analysis was performed for *TNFa* (**A**) and *IL6* (**B**). All data are expressed as mean ± s.d. from triplicate determinations. Source data are provided as a Source Data file.

A

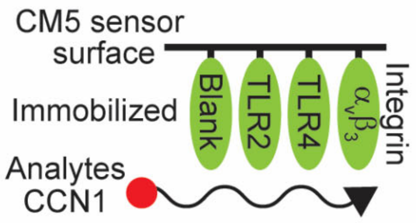

B

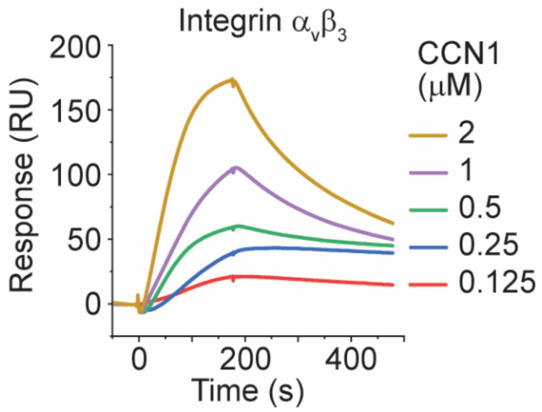

C

	ka ($M^{-1}s^{-1}$)	*kd* (s^{-1})	$\boldsymbol{K_D}$ **(nM)**
hTLR2	64600	0.01465	227
hTLR4	9959	0.00288	291
Integrin $\alpha_v\beta_3$	95380	0.01929	202

Supplementary Figure 13. Surface plasmon resonance (SPR) analysis of CCN1 binding to TLRs and integrin $\alpha_v\beta_3$. (**A**) A diagram of SPR analysis with CCN1 as analyte and TLR2, TLR4, and integrin $\alpha_v\beta_3$ as ligands immobilized on CM5 chip. (**B**) Sensorgrams showing the specific interaction between ligand (integrin $\alpha_v\beta_3$) and increasing concentration of CCN1 as analyte (CCN1; 0.025, 0.25, 0.5, 1, and 2 μM). (**C**) Kinetic values of CCN1 interaction with TLR2, TLR4, and integrin $\alpha_v\beta_3$ were obtained from the sensorgrams presented in **B** and in Fig. 8b,c.

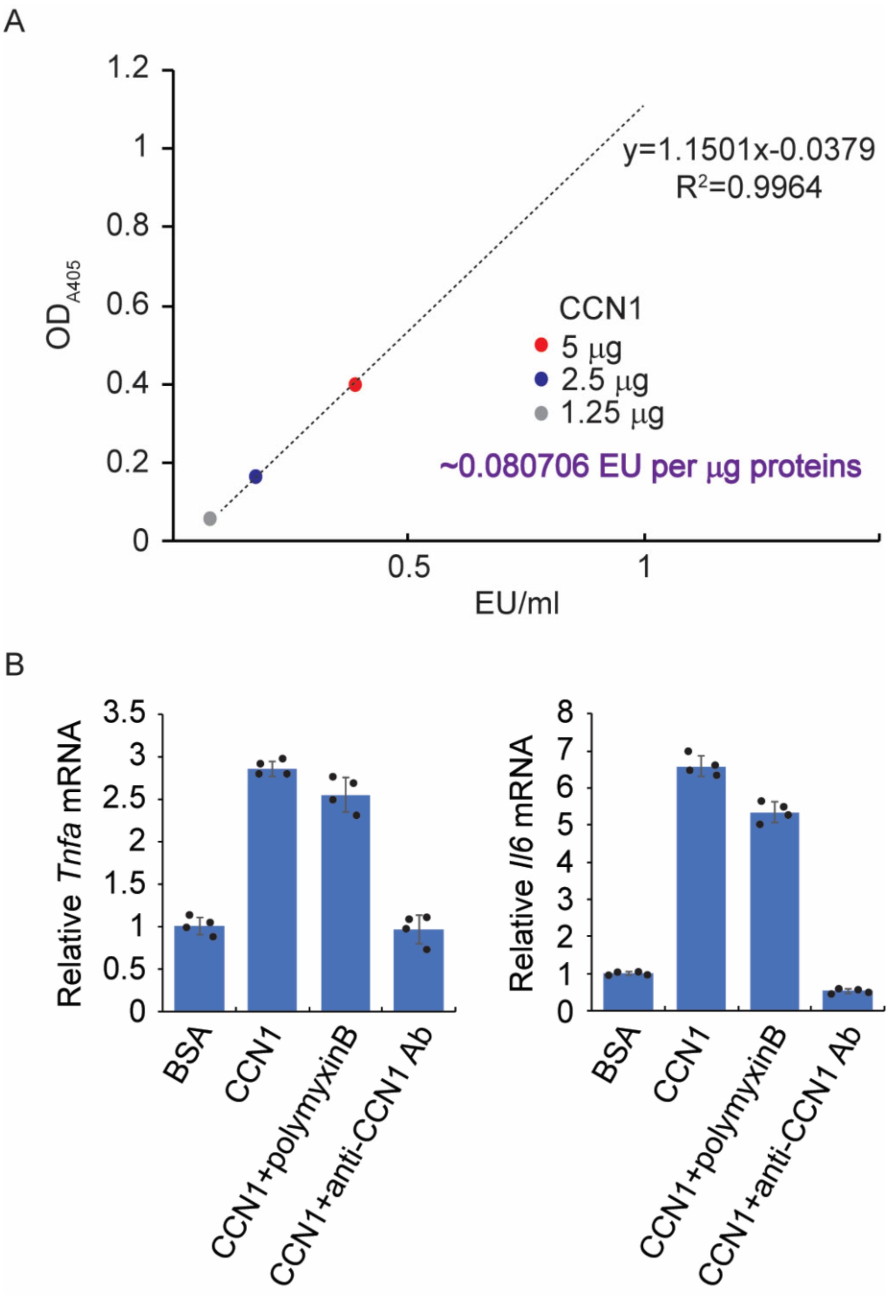

Supplementary Figure 14. Assays for endotoxin. (**A**) Recombinant CCN1 proteins were tested for endotoxin contamination using LAL assay. Dilutions of CCN1 (1.25, 2.5, and 5 μg) were measured for endotoxin and interpolated onto the standard curve generated using the manufacturers reagents, showing that the CCN1 protein prep contains ~0.08 EU per μg protein. A representative example is shown; other CCN1 preps yielded similar results. (**B**) CCN1 protein was passed through a polymyxin-B agarose column to remove any endotoxin; qPCR analysis showed that the resulting protein was still able to induce inflammatory gene expression. The activity of CCN1 proteins were also tested by antibody depletion using anti-CCN1 antibodies. All data in qPCR are expressed as mean ± s.d. from triplicate determinations. Source data are provided as a Source Data file.

Supplementary Table. 1. Target sequences of DsiRNAs

protein	Target sequence (5’ to 3’)
Integrin alpha 6	aaagggtaacatcaccttctattgcac
Integrin alpha v	gtcatatttagatatgatttctgccac
Integrin beta 3	attccttaactgcttgttctactactg
Integrin beta 5	ttccactagtgcatatgttgagccctg
Integrin alpha M	aggataaagtcaccttcatcaacac
Integrin beta 2	gctagagcatgagttatcataatca
