# Supplementary material for: CCN1 is an opsonin for bacterial clearance and a direct activator of Toll-like receptor signaling
Source: Nat Commun. 2020 Mar 6;11:1242. doi: 10.1038/s41467-020-15075-5 (PMC7060279; doi:10.1038/s41467-020-15075-5)
Supplement: Supplementary file 3 — Reporting Summary [file 41467_2020_15075_MOESM3_ESM.pdf]

nature research

Corresponding author(s): Lester Lau

Last updated by author(s): Jan 20, 2020

Reporting Summary

Nature Research wishes to improve the reproducibility of the work that we publish. This form provides structure for consistency and transparency in reporting. For further information on Nature Research policies, see Authors & Referees and the Editorial Policy Checklist .

Statistics

For all statistical analyses, confirm that the following items are present in the figure legend, table legend, main text, or Methods section.

n/a	Confirmed	
☐	☒	The exact sample size (*n*) for each experimental group/condition, given as a discrete number and unit of measurement
☐	☒	A statement on whether measurements were taken from distinct samples or whether the same sample was measured repeatedly
☐	☒	The statistical test(s) used AND whether they are one- or two-sided<br>*Only common tests should be described solely by name; describe more complex techniques in the Methods section.*
☒	☐	A description of all covariates tested
☒	☐	A description of any assumptions or corrections, such as tests of normality and adjustment for multiple comparisons
☐	☒	A full description of the statistical parameters including central tendency (e.g. means) or other basic estimates (e.g. regression coefficient) AND variation (e.g. standard deviation) or associated estimates of uncertainty (e.g. confidence intervals)
☐	☒	For null hypothesis testing, the test statistic (e.g. *F*, *t*, *r*) with confidence intervals, effect sizes, degrees of freedom and *P* value noted<br>*Give P values as exact values whenever suitable.*
☒	☐	For Bayesian analysis, information on the choice of priors and Markov chain Monte Carlo settings
☒	☐	For hierarchical and complex designs, identification of the appropriate level for tests and full reporting of outcomes
☒	☐	Estimates of effect sizes (e.g. Cohen's *d*, Pearson's *r*), indicating how they were calculated

Our web collection on statistics for biologists contains articles on many of the points above.

Software and code

Policy information about availability of computer code

Data collection	Microsoft Excel (Office 365), Image Pro Insight V.8.0 (Media Cybernetics), Adobe Illustrator CC2020, Adobe Photoshop CC2020
Data analysis	Microsoft Excel (Office 365), Origin 2019 (64-bit), CytoExpert (Beckman Coulter), Biacore T200 evaluation software V3.0 (Biacore)

For manuscripts utilizing custom algorithms or software that are central to the research but not yet described in published literature, software must be made available to editors/reviewers. We strongly encourage code deposition in a community repository (e.g. GitHub). See the Nature Research guidelines for submitting code & software for further information.

Data

Policy information about availability of data

All manuscripts must include a data availability statement. This statement should provide the following information, where applicable:

- Accession codes, unique identifiers, or web links for publicly available datasets
- A list of figures that have associated raw data
- A description of any restrictions on data availability

Data supporting the findings of this work are available within the paper and the Supplementary Information files. A reporting summary is available as a Supplementary Information file. The raw datasets generated and analyzed for SPR study are available from the corresponding author upon request. The source data for Figs. 4c, 4e, 5b, 5d, and 5e, and Supplementary Fig.10B are provided in a Source Data

Field-specific reporting

Please select the one below that is the best fit for your research. If you are not sure, read the appropriate sections before making your selection.

Life sciences study design

All studies must disclose on these points even when the disclosure is negative.

Sample size	No sample size calculation was performed beforehand. Sample sizes were chosen according to established practice in the field
Data exclusions	No data point has been excluded.
Replication	Studies were done with CCN1 proteins from the lab and replicated with CCN1 proteins obtained from two commercial sources with distinct cellular origins and different purification methods.
Randomization	N/A
Blinding	N/A

Reporting for specific materials, systems and methods

We require information from authors about some types of materials, experimental systems and methods used in many studies. Here, indicate whether each material, system or method listed is relevant to your study. If you are not sure if a list item applies to your research, read the appropriate section before selecting a response.

Materials & experimental systems

n/a	Involved in the study
☐	☒ Antibodies
☐	☒ Eukaryotic cell lines
☒	☐ Palaeontology
☐	☒ Animals and other organisms
☒	☐ Human research participants
☒	☐ Clinical data

Methods

n/a	Involved in the study
☒	☐ ChIP-seq
☐	☒ Flow cytometry
☒	☐ MRI-based neuroimaging

Antibodies

Antibodies used	anti-CCN1 Ab (from house; 1:2000 dilution for solid phase binding and 1:100 for flow cytometry); polyclonal anti-S. aureus (ab20920; 1:2000 dilution for solid phase binding and 1:100 dilution for IHC), anti-P. aeruginosa (ab68538; 1:500 dilution for solid phase binding), anti-S. pneumonea (ab20429; 1:2000 dilution for solid phase binding), and monoclonal anti-S. Typhimurium antibodies (ab8274; 1:1000 dilution for solid phase binding) (Abcam); anti-hTLR2 (MAB2616, 1:100 dilution for solid phase binding and 1:1000 dilution for dot blot), anti-hTLR4 (AF1478; 1:100 dilution for solid phase binding and 1:1000 dilution for dot blot), or anti-hCD14 antibodies (MAB3832; 1:100 for solid phase binding) (R&D systems); monoclonal anti-iC3b antibody (EMD Millipore, MABF972; 1:200 dilution); allophycocyanin (APC)-conjugated goat anti-rabbit IgGs (Life Technologies Co.; A10931, 1:250 dilution;) or APC-conjugated rat anti-mouse IgGs (Life Technologies Co.; 17-4015-82, 1:500 dilution); Alexafluor488-conjugated anti-rabbit IgG (Invitrogen, 1:500 dilution)
Validation	anti-CCN1 antibody was validated in Western blot analysis, immunohistochemistry and flow cytometry. Each antibody validation can be found in each company's web site.

Eukaryotic cell lines

Policy information about cell lines

Cell line source(s)	All cell lines were obtained from American Type Culture Collection.
Authentication	For L929 cells, morphology and ability of conditioned media for macrophage differentiation were used for authentication. For THP.1 cells, morphology and PMA-induced differentiation was used.
Mycoplasma contamination	All cell lines were tested negative for Mycoplasma contamination using commercial kit.
Commonly misidentified lines (See ICLAC register)	none

Animals and other organisms

Policy information about studies involving animals; ARRIVE guidelines recommended for reporting animal research

Laboratory animals	Ccn1D125A/D125A and Ccn1DM/DM knock-in mice, a svJ129-C57BL/6J mixed background and backcrossed to the C57BL/6J strain 6 and 11 times, both male and female, 10-12 weeks of age with similar body weight (25-28 g); Ccn1ΔMyeloid mice were generated by crossing Ccn1flox/flox with myeloid-specific Cre deleter stain, LysM-Cre (004781, Jackson Laboratory); Tlr2-/- (004650), Tlr4-/- (029015), and Myd88-/- mice (009088) were from the Jackson Laboratory
Wild animals	The study did not involve Wild animals.
Field-collected samples	The study did not involve samples collected in the field.
Ethics oversight	Animal protocols were approved by the Institutional Animal Care and Use Committee of The University of Illinois at Chicago

Note that full information on the approval of the study protocol must also be provided in the manuscript.

Flow Cytometry

Plots

Confirm that:

- [x] The axis labels state the marker and fluorochrome used (e.g. CD4-FITC).
- [x] The axis scales are clearly visible. Include numbers along axes only for bottom left plot of group (a 'group' is an analysis of identical markers).
- [ ] All plots are contour plots with outliers or pseudocolor plots.
- [x] A numerical value for number of cells or percentage (with statistics) is provided.

Methodology

Sample preparation	S. aureus collected in the logarithmic phase were heat-killed in 80°C for 30 min and resuspended in PBS buffer containing 2 mM EDTA and 0.5% BSA. S. aureus (10^8 CFU per ml)
Instrument	CytoFLEX S flow cytometer (APC channel, Beckman Coulter)
Software	CytoExpert software
Cell population abundance	more than 10,000 events were measured
Gating strategy	All events were gated on FSS vs SCS to sort target population (P1), followed by SCS vs SCS-width to obtain the population without aggregated bacterial particles (P2). To determine the percentage of APC-positive population, the number of events were recorded at specific fluorescence intensity. No treatment with antibodies was used to define APC-positive population

- [x] Tick this box to confirm that a figure exemplifying the gating strategy is provided in the Supplementary Information.
